# Meta-analytic method reveal a significant association of the*BDNF* Val66Met variant with smoking persistence based on a large samples

**DOI:** 10.1038/s41397-019-0124-y

**Published:** 2019-12-02

**Authors:** Hailong Zhao, Shuicai Xiong, Zhiwei Li, Xuebiao Wu, Lijuan Li

**Affiliations:** 1 Department of Pathophysiology, Zunyi Medical University, Zunyi, 563000 China; 2 People’s hospital of Jinxian County, Nanchang, 331700 China; 3 The Second Affiliated Hospital of Nanchang University, Nanchang, 331700 China

**Keywords:** Genetic markers, Risk factors

## Abstract

Although numerous genetic studies have reported the link between Val66Met in *BDNF* gene with smoking, the findings remain controversial, mainly due to small-to-moderate sample sizes. The main aim of current investigation is to explore whether the variant of Val66Met has any genetic functions in the progress of smoking persistence. The Val-based dominant genetic model considering Val/* (namely, Val/Val + Val/Met) and Met/Met as two genotypes with comparison of the frequency of each genotype in current smokers and never smokers. There were seven genetic association articles including eight independent datasets with 10,160 participants were chosen in current meta-analytic investigation. In light of the potent effects of ethnicity on homogeneity across studies, we carried out separated meta-analyses according to the ancestry origin by using the wide-used tool of Comprehensive Meta-analysis software (V 2.0). Our meta-analyses results indicated that the Val66Met polymorphism was significantly linked with smoking persistence based on either all the chosen samples (*N* = 10,160; Random and fixed models: pooled OR = 1.23; 95% CI = 1.03–1.46; *P* value = 0.012) or Asian samples (*N* = 2,095; Fixed model: pooled OR = 1.25; 95% CI = 1.01–1.54; *P* value = 0.044; Random model: pooled OR = 1.25; 95% CI = 1.001–1.56; *P* value = 0.049). No significant clue of bias in publications or heterogeneity across studies was detected. Thus, we conclude that the Val66Met (rs6265) variant conveys genetic susceptibility to maintaining smoking, and smokers who carry Val/* genotypes have a higher possibility of maintaining smoking than those having Met/Met genotype.

## Introduction

Cigarette smoking, a chronic and complex brain-related disorder, has been documented to lead to many diseases, including various cancers [1–3]. Notably, smoking conveys highly susceptibility to lung cancer, ranging from fivefold to tenfold. However, the smoking prevalence is still high or increasing in some Asian countries. Recently, an authoritative report from World Health Organization [4] demonstrated that there were about six million deaths worldwide each year resulting from smoking. In China, the largest tobacco-consuming country in the world, suffering a huge health hazard threating from cigarette smoking, which leads to ~1.4 million people to die in the year of 2010 [5–7]. Badly, the number of smoking-related deaths is estimated to reach to two million in the year of 2030 and about three million in the year of 2050 if the patterns of smoking in China are unchanged [6–8]. Thus, it is necessary to develop powerful and effective approaches for promotion of smoking cessation and prevention of smoking initiation.

Twin- and family-based genetic studies [9–13] have documented various behaviors relevant to smoking, including the initiation of smoking, persistence in smoking, dependence of nicotine, attempt to stop smoking, and cessation, are affected by both environmental factors and genetic components. In total, the average heritability of dependence of nicotine is 0.56 in both male and female smokers [9]. Additional studies have reported a similar level of heritability for smoking initiation and cessation [12, 13]. Thus, a large and growing interests in exploring the risk of genetic components for smoking and subsequent nicotine dependence. Many studies based on candidate genes and several large genome-wide association studies (GWAS) have reported a great number of variants relevant to smoking behaviors [14–19]. One of the most promising results in GWASs is genetic variants mapped in the *CHRNA5-A3-B4* gene cluster on the chromosome of 15q24–25.1 region are remarkably linked with nicotine dependence [16, 19].

Genetics-based linkage and association studies have implicated the functions of genetic components in the pathogenesis of smoking-related behaviors, including the effect on the dopamine-related reward circuit [14]. Nicotine could activate dopamine-related nerve fibers in the mesolimbic-reward pathway and increase the concentrations of extracellular dopamine that are higher than those stimulations from sex and food [20, 21]. Since the dopaminergic reward circuit is extensively involved in the pathogenesis of smoking behaviors, various genes in the pathway have been extensively chosen for good candidates in smoking-related genetic association studies. Recent studies have demonstrated the neurotrophic factor family protein of brain-derived neurotrophic factor (BDNF), a potent dopamine modulating protein, is considered to involve in the effects of nicotine on enhancing cognitive functions, which potentially modulate nicotine reward [22, 23].

BDNF, encoded by *BDNF* gene in the chromosome 11p13–15, shows highly expression in the mammalian brain, and involves in regulation of multiple biological functions of neurons, such as neuron development, regeneration, survival, and maintenance [24]. BDNF is a required element for modulating the neuron development and molecular regulation of dopaminergic reward pathways [25–27]. Animal and human studies have reported that cognitive stimulation, antidepressant treatment, and physical activity improve BDNF secretion, whereas mood disorders and stress reduce its secretion [28]. These previously reported findings showed that BDNF might regulate the responsiveness of dopamine in such a vital way that may relevant to the etiology or treatment of several conditions implicating dopamine. Earlier studies [29] have documented that chronic nicotine could increase *BDNF* expression in the rat hippocampus, whereas acute nicotine prominently decreases the gene expression of *BDNF*. Genome-wide linkage investigations have suggested that the chromosome 11p13 region is likely to harbor genes conferring susceptibility to nicotine dependence [30]. Recent genetics-based association studies [19, 23, 31, 32] have demonstrated that variants in *BDNF* implicated in vulnerability to smoking behaviors.

In particular, the genetic variant of Val66Met (i.e., rs6265) has been investigated extensively to influences the BDNF system. The functional polymorphism of Val66Met was observed to change BDNF intracellular packaging and trafficking, which affects the activity-dependent secretion of BDNF protein [33]. Since Beuten et al. first [34] found a significant link between genetic variant in *BDNF* and dependence of nicotine in male smokers based on European-American origin, a growing number of genetics-based association researches [23, 35–42] have been performed to reveal the genetic correlation of rs6265 with cigarette smoking-related traits. Nevertheless, these results are still equivocal. Furthermore, more relevant studies are essential for better determining the functional role of rs6265 in smoking behaviors and whether harboring the variant of rs6265 may have clinical therapeutic implications. To our best knowledge, no meta-analysis has performed a combined analysis of the effect of rs6265 in *BDNF* on smoking persistence. Thereby, we conducted a meta-analysis based on a large-scale sample sizes of existing studies for the association between rs6265 in *BDNF* and smoking persistence.

## Materials and methods

### Effective search strategy and stringent inclusion criteria

We interrogated the public database of NCBI PubMed (https://www.ncbi.nlm.nih.gov/pubmed) for studies with regard to the association of variants in*BDNF* with smoking behaviors prior to June 25, 2018. The searching keywords used were “susceptibility”, “polymorphism”, “genetics”, “variant”, “*BDNF*”, “brain-derived neurotropic factor”, “smoking”, “nicotine”, “cigarette”, and “tobacco”. Abstracts were tested for possible related papers in consistent with the normal criteria for inclusion and exclusion proposed by Moher et al. [43]. The references cited in these full text papers were hand-checked for underlying other-related publications missed by the original search. Duplication studies were dumped and articles reported in previous data were removed.

### Data extraction

According to the “Quality of Reporting of meta-analysis and PRISMA guidelines” [43], nine studies [23, 35–42] were read carefully for potent eligible dataset according to a strictly systematic and comprehensive review (see Supplementary Fig. S1 for details). Only studies or datasets meeting the five strict criteria as following were chosen: (1) a case-control-based studies (namely, family-based studies were abandoned); (2) a peer-reviewed publication; (3) data were independent from other previous studies; (4) raw genotype or allele frequency could be accessed for use; and (5) sufficient data could be employed to reckon an odds ratio (OR) and 95% confidence interval (CI). For each chosen study, two authors (Wu Xubiao and Hailong Zhao) used a standardized forms to extract the following data: author names and publication years, paper based on English or based on other kinds of languages, used samples’ ancestry, types of the chosen studies, ethnicity, the number of participants in each chosen study, gender ratio, *P* values of Hardy–Weinberg equilibrium (HWE), criteria of diagnosis for defining smoking phenotype, and the count of subjects with distinct smoking status stratified by the genotypes of rs6265.

### Classification of phenotypes and genotypes

As documented in previous studies [15, 18, 44], we defined the elements of smoking behaviors as comparing the status of smoking: (1) use ever smoking versus (vs.) never smoking to assess initiation of smoking, (2) use current smoking vs. never smoking to assess maintaining smoking (i.e., smoking persistence), and (3) use current smoking vs. ex-smoking to assess smoking cessation. In the accepted studies, the data for smoking was mainly concentrated on smoking persistence. Thus, we carried out our meta-analysis for the association of Val66Met genotypes in *BDNF* under a dominant model (Val/Val and Val/Met genotypes vs. Met/Met genotypes) with smoking persistence.

### Statistical analysis

Firstly, we combined all datasets from the included articles to conduct an overall meta-analysis. Further, we performed separated meta-analyses through taking into account the underlying influence of different race: Asian ancestry and Caucasian ancestry. Since there is only one study for African ancestry, no stratified meta-analysis for this race. All meta-analyses were conducted by employing the wide-used tool of Comprehensive Meta-analysis software package (V. 2.0; Biostat Inc., Englewood, NJ). The significance of a combined OR is calculated by a *Z*-score examination, and *P* value < 0.05 is thought to be significant.

We used both random and fixed models for meta-analyses. With respect the random model based on DerSimonian and Laird methods [45], which assumes that the heterogeneity across distinct chosen studies is attributed to variation within- and between-study, the effect size of each study was computed to create a combined OR and 95% CI. The effect size of each single study using the fixed model was combined with the use of Mantel–Haenszel methods [45], which assumes that genotype effect is constant across different researches and the observed variation is attributed to chance of random. Compared with the fixed model, the random model that generating a wider CI is more conservative. Thus, the fixed model appears to be more encouraged when no heterogeneity across studies exists; otherwise, the random model should be acceptable.

We used both Cochran’s *Q* and*I*^2^ test [46] to evaluate the potential heterogeneity between different chosen studies. The across-all-studies heterogeneity is of significance when *P*_*Q*_ < 0.05, and further it can be assessed by the value of *I*^2^ (*I*^2^ = *Q* − df/*Q*), which is employed for determining the percentage of evaluated variation across different chosen studies resulting from heterogeneity instead of that by chance of random. For a typical example, when *I*^2^ value is equal to 50%, the outcomes from meta-analyses can inflate evaluated variation percentage not explained by genotype to 50% [15, 18]. Further, we also utilized two kinds of approaches of funnel-shaped distribution and Egger-regression test to assess publication bias. Funnel plot uses a method of linear regression to visualize the funnel-shaped distribution asymmetry based on the natural logarithm of the OR. When several chosen studies were out of the funnel distribution, the plot tends to be asymmetrical, indicating there exists a possibility of bias in publication.

## Results

### Basic characteristics on the chosen studies

In current study, we searched ten studies reported between 2007 and 2017 (Supplementary Table S1). All these chosen studies contain a total of 11,348 subjects, ranging from 149 to 3404 samples in each study. There are two main populations based on Asian ancestry and Caucasian ancestry. Among these studies, different sex ratios were detected (Table 1). Except for the study of Zhang et al. [37], the genotypes of all studies were HWE (Supplementary Table S1). Since other three typical genetic-based models, i.e., allelic, additive, and recessive genetic model, showed no evidence of a significant association between Val66Met and smoking persistence (data not included), we thus adopted a genetic model of Val/* genotype dominant for all the subsequent analyses. There are seven eligible studies with a total of 10,160 samples for meta-analysis (Table 1). Genotype distributions of Val/* (i.e., Val/Val and Val/Met genotypes) of these eight studies are shown in Table 1.

**Table 1:** Characteristics of each study chosen in current meta-analysis (*N* = 10,160 participants)

Study name	Publication year	Sample Size	% Caucasian	% male	Current smoking			Never smoking			Val/* genotype frequency (%)
					Val/Val	Val/Met	Met/Met	Val/Val	Val/Met	Met/Met	
Lang et al.	2007	253	100	48.4	67	41	6	105	30	4	96.0
Wang et al.	2007	149	0 (100% Han Chinese)	100	30	51	20	12	20	16	75.8
Montag et al.	2008	554	100	35.3	90	47	6	264	136	11	96.9
Landi et al.	2009	2238	100	NA	879	521	62	458	277	41	95.4
Zhang et al.	2012	628	0 (100% Han Chinese)	100	81	177	64	74	156	76	77.7
Zhang et al.	2015	1318	0 (100% Han Chinese)	100	215	456	173	114	253	107	78.8
Jiang et al. (EA)	2017	1616	100	49.3	623	235	17	508	210	23	97.5
Jiang et al. (AA)	2017	3404	0 (100% African American)	51.8	1543	121	1	1636	100	3	99.9

### Meta-analysis based on all included studies

**Fig. 1:**
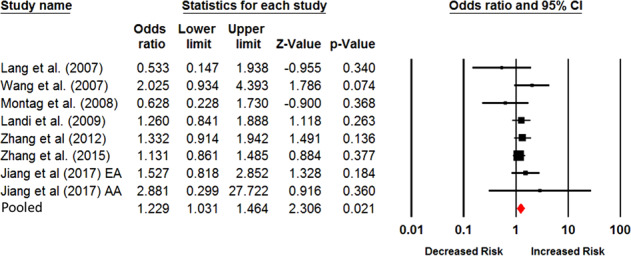
Forest plot shows the results from current meta-analysis for the association of the polymorphism of Val66Met in *BDNF* with smoking persistence based on all chosen studies. The specific odds ratio, lower limit, upper limit, *Z*-score, and*P* value of each individual study are shown by rows. The central vertical solid line represents the value of ORs. The OR of null hypothesis is assumed to be equal to 1. We used the horizontal and square bar to represent the 95% CI and OR of each study. The pooled OR is computed in the fixed model and underneath of the forest plot with the use of diamond symbol for representation

We first carried out an overall meta-analysis to reveal whether the link of Val66Met genotypes in *BDNF* with maintaining smoking by incorporating all the chosen datasets. The dataset from the chosen studies reported by Jiang et al. [35] was stratified into two datasets based on Asian and Caucasian ancestry (Table 1). The pooled OR was 1.23 (*P* value = 0.021) with the 95% CI from 1.03–1.46 in both the fixed and random models (Fig. 1 and Table 2), suggesting that the Val/* genotypes convey a higher susceptibility to smoking persistence. The Cochran’s *Q* and *I*^2^ test demonstrated no evidence of significant heterogeneity across all the chosen studies existed (*I*^*2*^ = 0.0; *P*_*Q*_ = 0.49).

**Table 2:** Results from the meta-analysis for smoking persistence stratified by different datasets

Dataset of meta-analysis	Cohorts (*n*)	Sample size (*N*)	Estimate of Heterogeneity		Fixed-effects model			Random-effects model			*B*^(d)^
			*I*^2^ (%)	*P* (Q)	Pooled OR	95% CI	*P* (Z)	Pooled OR	95% CI	*P* (Z)	*P* (*B*)
All samples^a^	*n* = 8	*N* = 10,490	0.0	0.49	1.23	1.03–1.46	0.021	1.23	1.03–1.46	0.021	0.71
Asian samples^b^	*n* = 3	*N* = 2,095	5.6	0.35	1.25	1.01–1.54	0.044	1.25	1.001–1.56	0.049	0.08
Caucasian samples^c^	*n* = 4	*N* = 4,991	18.9	0.296	1.18	0.86–1.61	0.31	1.14	0.78–1.66	0.51	0.2

Current results were based on Val/* dominant model

^a^Meta-analysis of all samples from all chosen studies

^b^Meta-analysis of all Asian samples from chosen studies

^c^Meta-analysis of all Caucasian samples from chosen studies

^d^Egger’s regression test for publication bias

### Meta-analysis based on different ethnicities

**Fig. 2:**
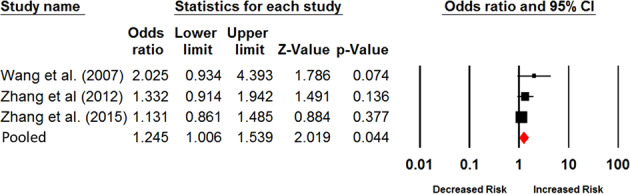
Forest plot shows the results from current meta-analysis for the association of the polymorphism of Val66Met in *BDNF* with smoking persistence based on Asian population-based studies. The specific odds ratio, lower limit, upper limit, *Z*-score, and *P* value of each individual study are shown by rows. The central vertical solid line represents the value of ORs. The OR of null hypothesis is assumed to be equal to 1. We used the horizontal and square bar to represent the 95% CI and OR of each study. The pooled OR is computed in the fixed model and underneath of the forest plot with the use of diamond symbol for representation

By considering the Val/* genotypes frequency differences between different populations, we conducted two stratified meta-analyses, incorporating the datasets depended on Asian population and Caucasian population. With respect to the Asian ancestry, the combined OR was 1.25 (95% CI = 1.01–1.54; *P* = 0.044) in the fixed model (Fig. 2). Similar with the outcomes from overall meta-analysis, no evidence of significant across-study heterogeneity was observed in Asian population (*I*^*2*^ = 5.6; *P*_*Q*_ = 0.35; see Table 2). When the meta-analysis for Asian population conducted in the random model, the association is still significant with the pooled OR of 1.25 (95% CI = 1.001–1.56; *P* = 0.049). Although there was no evidence of significant heterogeneity between studies of Caucasian population (*I*^*2*^ = 18.9; *P*_*Q*_ = 0.296), no significant association was detected (Table 2 and Supplementary Fig. S2).

### Sensitivity-based and accumulative-based analysis for reliability of current meta-analysis

**Fig. 3:**
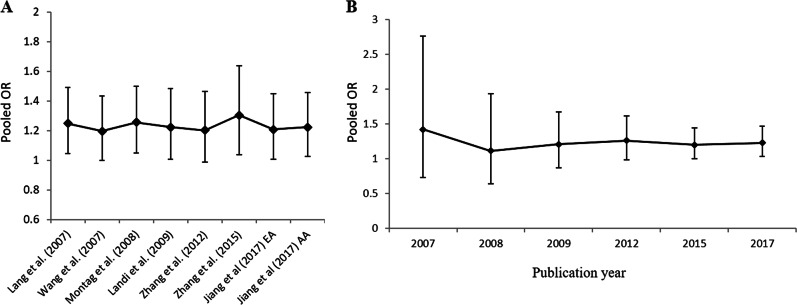
Plots of sensitivity-based and accumulative-based analysis results for the meta-analyses combined all chosen studies. **a** The *Y*-axis represents the combined OR, and the *X*-axis for each single study left out in sequence from the chosen articles. The diamond symbols stand for the combined OR, and the bottom and top horizontal bars represent the 95% confidence intervals (CIs). **b** The pooled OR of the rs6265 polymorphism for smoking persistence was shown against years of publication among all chosen studies. The *Y*-axis represents the pooled OR and the *X*-axis for the years of studies published relative to the pooled OR. The diamond symbols show that the pooled OR, and each vertical line with horizontal bars labels the 95% CI

A sensitivity-based analysis was carried out for datasets from all the chosen articles in the fixed model to test whether the detected effect of Val66Met variant on smoking persistence was significantly affected by kicking out one independent article each time. As presented in Fig. 3, the combined ORs fluctuated faintly ranging from 1.196 to 1.303, suggesting our current findings from meta-analytic approach were not prominently impacted by any single dataset from chosen study. All calculated P values for sensitivity-based test were detected to be statistically significant (Fig. 3a and Supplementary Table S2). To determine whether Val66Met polymorphism showing significant association with smoking persistence alters with the increase of years of publication, we also conducted an accumulative-based analysis for all the datasets from our current chosen studies in the fixed model. The combined ORs reduced from 1.422 in 2007 to 1.207 in 2009, and went to relatively stable after the year of 2009 to now (Fig. 3b). Please refers to Supplemental Table S3 for the specific*P* values and other detailed relevant information.

### Assessment of possible bias in publication

**Fig. 4:**
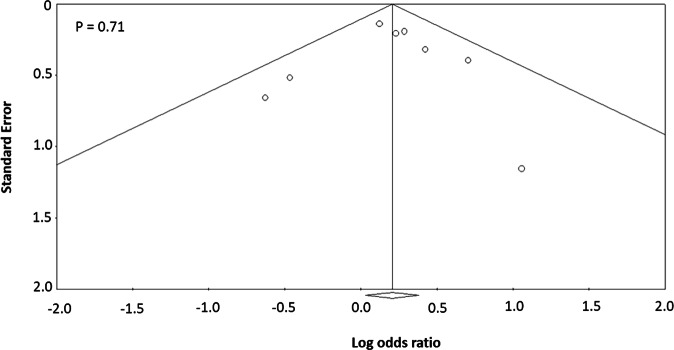
Funnel plots of publication biases of Asian population-based studies. Here, *Y*-axis stands for the standard error of the log OR and *X*-axis represents the log OR. Each dot represents an individual study

For all these performed meta-analyses in the current investigation, we employed the approaches of Egger-regression analysis and funnel-shaped distribution to evaluate the existing bias in publication. As shown in Table 2, all the calculated*P* values from the Egger-regression analysis were >0.05, suggesting that there existed no significant evidence of publication bias in all three meta-analyses performed in the present investigation. Consistently, the funnel-shaped plots for meta-analyses based on all chosen studies, Asian population, and Caucasian population tend to be of symmetry with no study deviated out of the funnel-shaped plot (Fig. 4, Supplementary Figs. S3 and  S4), which provides further supportive evidence of no publication bias for all these conducted meta-analyses.

### Functional analysis of rs6265 polymorphism in BDNF

**Fig. 5:**
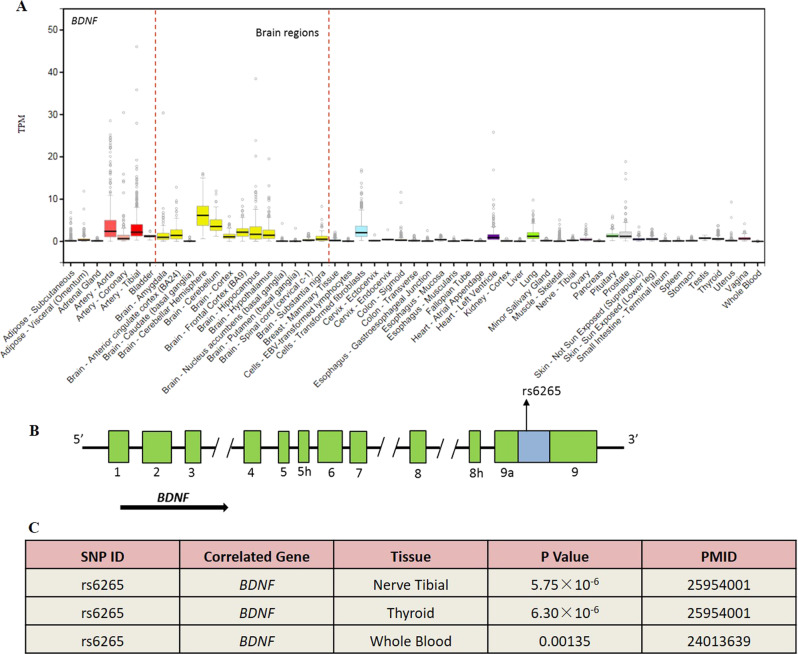
The *BDNF* gene expression in different 53 human tissues and rs6265-eQTL analysis. **a** For BDNF gene expression. The source of expression data were attained from GTEx analysis release V7 (dbGaP Accession phs000424.v7.p2). Expression values are shown in transcripts per million (TPM). Box plots are presented as median, 25th and 75th percentiles; points are shown as outliers if the value above or below 1.5 times the interquartile range. **b** The Human *BDNF* gene structure. The 10 introns are presented as black lines and the 11 exons as green boxes. Only the 3’ exon 9 is coding and generates the polypeptide of BDNF. The blue box represents the region of exon 9 coding for the pro-BDNF protein. The functional rs6265 polymorphism is located at the position of 196 in the genomic region encoding the protein of pro-BDNF [31, 58]. **c** rs6265-eQTL analysis. The cis-acting SNP-eQTL analyses in different human tissues was based on a web-based tool of Haploreg v4.1 (https://pubs.broadinstitute.org/mammals/haploreg/haploreg.php)

Consistent with previous evidence [24], we observed the *BDNF* gene is highly expressed in human brain tissues based on the web-based database of GTEX PROTAL (https://gtexportal.org/home/). The nonsynonymous Val66Met (C196T) polymorphism (rs6265) is located at the position of 196 in the genomic region encoding pro-BDNF protein (Fig. 5b). To further explore the cis-regulatory effects of rs6265 polymorphism on the expression of *BDNF* gene, we performed cis-acting eQTL analyses in human tissues by using a web-based tool of Haploreg v4.1. We observed that the polymorphism of rs6265 showed prominent allele-specific mRNA expression of*BDNF* in nerve tibial tissue (*P* = 5.75 × 10^−6^), thyroid tissue (*P* = 6.30 × 10^−6^) and whole blood samples (*P* = 0.00135) (Fig. 5c).

## Discussion

In current investigation, we first conducted a meta-analysis to identify the association between Val66Met (rs6265) in *BDNF* and smoking persistence, and detected a prominent effect of Val66Met polymorphism on smoking persistence based on a large-scale samples. Since there exist no significant evidence of heterogeneity across all chosen studies or publication bias identified, we thus concluded that the Val allele play a crucial role in the pathology of the persistence of smoking, suggesting that carriers of Val/Val and Val/Met genotypes have a higher likelihood of maintaining smoking than individuals carrying the homozygous genotype of Met/Met.

The dopaminergic reward system functionally response to the stimulation of nicotine is regulated by BDNF protein, which could regulate dopamine D3 receptor (DRD3) expression through a regulation system of stimulating the function of the DRD1 [47]. The encoding *BDNF* gene, located on chromosome 11, has been well-reported to be correlated with substance abuses and psychiatric diseases, such as nicotine dependence [19, 31], alcohol dependence [31, 48], schizophrenia [49], personality [28], and attention deficit hyperactivity disorder [50]. The well-studied polymorphism of Val66Met, located in the region of exon 9 that codes for the pro-BDNF, was observed to change BDNF intracellular packaging and trafficking, which affects the activity-dependent secretion of BDNF protein [33]. The rs6265 variant has been demonstrated to be functionally linked with the impaired function of memory and hippocampal [51] and increased anxiety-related behaviors [52]. Consistently, multiple lines of evidence have documented that the genetic variant of rs6265 is significantly associated with various psychiatric diseases, including schizophrenia, bipolar, and eating disorders [53, 54]. Furthermore, a number of genetic researches have reported a potential association between the common Val66Met variant and susceptibility to smoking-related phenotypes [23, 35–42]. For example, a large-scale GWAS [19] identified the polymorphism of rs6265 was significantly associated with smoking initiation (OR = 1.06, 95% CI 1.08–1.18,*P* = 3.6 × 10^−8^). However, the pattern of the results for smoking persistence is inconsistent, which primarily because of the small sample size or sampling bias in each investigation. It remains an open question whether the genetic variant of rs6265 has an effect on smoking persistence. The meta-analytic method is extensively thought to be an effective tool for improving the power of our statistical analysis through combining various studies on the same topic.

In view of recessive, additive, and allelic genetic models have no significant results, the Val-dominant model was mainly applied in current meta-analytic investigation. The rs6265 polymorphism was detected to be significantly correlated with maintaining smoking based on all chosen samples and Asian ancestry-based samples. As for Caucasian population, we did not detect any evidence for the link. The allele frequencies of Val allele of rs6265 variant show highly differences across different population, with Val allele estimated to be ~78.4% in Caucasian population but only about 51.5% among Asian populations, which is in consistent with the findings reported by previous articles [28, 55]. As such, the different frequencies of rs6265 allele may contribute to the nonsignificant association of smoking persistence among Caucasian population. However, we observed on significant clue of bias in publication or between-study heterogeneity in all the present meta-analyses. Further, no study fell noticeably out of the funnel-shaped distribution. After applying of the method of Duval and Tweedie trim and fill by adding missing negative studies, our meta-analyses-based results remain to be significant. In addition, from sensitivity-based and accumulative-based analysis, the findings from meta-analysis based on all the chosen studies showed that the combined OR was not substantially vulnerable to any single study and gradually tended to steady as the years of relevant publications increased. Our interesting results offer strong evidence that the effect of rs6265 polymorphism contribute susceptibility to maintaining smoking.

Although a significant association between rs6265 and smoking persistence was observed, the findings of current meta-analytic investigation should be cautiously explained in light of underlying flaws as listed in the following. First, smoking is a common brain disorder with a complex etiology influenced by multiple factors and commonly show a comorbidity with other neuropsychiatric disorders or drug addictions, which appears to have a highly likelihood to share common genetic susceptibility components in the dopamine-related reward circuit. Further, the vast majority of the chosen studies did not offer relevant data that allows us to include or exclude subjects with comorbidity diseases in the selected samples. Thus, these comorbidity diseases in included smokers or never smokers might confuse the results of current meta-analysis. Second, there existed distinct gender ratios of subjects among these chosen articles, which might contribute to the existing limitations, as indicated by many previous reported studies [15, 18, 56, 57]. Third, in the present study, we did not compute the inter-rater-reliability for choosing published studies, which might cause some selecting biases. Nevertheless, if such bias existed, it would be minimized by using two authors independently reviewed all these published studies. Finally, due to there exist a deficient number of published articles on other variants, we could not test the biological interactions of gene by gene, which confer risk to the etiology of addictions or addictive behaviors, including smoking persistence. For example, Terracciano et al. [28] have documented that *BDNF* Val66Met show genetic interaction with 5-HTTLPR variant in the serotonin transporter gene. They demonstrated that individuals with BDNF Met variant and 5-HTTLPR LL have a higher scores of neuroticism, but with BDNF Val variant and 5-HTTLPR LL have a lower scores of neuroticism.

In sum, our current meta-analyses show the Val66Met variant has a moderate effect on smoking persistence in a large-scale samples. Compared with individuals with Met/Met genotype, individuals with Val/* genotypes have a 23% greater possibility of maintaining smoking, which suggests that the dopamine-relevant functions of Val66Met variant is implicated in mediating the biological process of smoking persistence. More related molecular experiments are needed to explore the biological function and regulation mechanism of the rs6265 variant on smoking persistence and reveal the genetic interactions with other genes. Current investigation advances our understanding of genetic components underlying smoking persistence, and would contribute to the development of effective strategies for smoking cessation.

## Supplementary information

Supplemental Tables

Supplemental Figures
