# Supplementary material for: Meta-analytic method reveal a significant association of theBDNF Val66Met variant with smoking persistence based on a large samples
Source: Pharmacogenomics J. 2019 Dec 2;20(3):398–407. doi: 10.1038/s41397-019-0124-y (PMC7253357; doi:10.1038/s41397-019-0124-y)
Supplement: Supplementary file 1 — Supplemental Tables [file 41397_2019_124_MOESM1_ESM.docx]

Supplemental Table S1. Characteristics of each study chosen in current meta-analysis (N=11,348 participants)

| Study Name | Publication Year | Sample Size | % Caucasian | % Male | Current Smoking | | | Never Smoking | | | Hardy-Weinberg Equilibrium (HWE) |
| --- | --- | --- | --- | --- | --- | --- | --- | --- | --- | --- | --- |
|  |  |  |  |  | Val/Val | Val/Met | Met/Met | Val/Val | Val/Met | Met/Met |  |
| Lang et al. | 2007 | 253 | 100 | 48.4 | 67 | 41 | 6 | 105 | 30 | 4 | 0.74 |
| Wang et al. | 2007 | 149 | 0 (100% Han Chinese) | 100 | 30 | 51 | 20 | 12 | 20 | 16 | 0.86 |
| Montag et al. | 2008 | 554 | 100 | 35.3 | 90 | 47 | 6 | 264 | 136 | 11 | 0.52 |
| Landi et al. | 2009 | 2,238 | 100 | NA | 879 | 521 | 62 | 458 | 277 | 41 | 0.51 |
| Zhang et al. | 2012 | 628 | 0 (100% Han Chinese) | 100 | 81 | 177 | 64 | 74 | 156 | 76 | 0.31 |
| Suriyaprom et al. | 2013 | 311 | 0 (%100 Thai Population) | 100 | 56 | 144 | | 36 | 75 | | 0.525 |
| Jamal et al. | 2015 | 877 | 100 | 33.6 | 357 | 207 | | 211 | 102 | | 0.3 |
| Zhang et al. | 2015 | 1,318 | 0 (100% Han Chinese) | 100 | 215 | 456 | 173 | 114 | 253 | 107 | 0.02 |
| Jiang et al. (EA) | 2017 | 1,616 | 100 | 49.3 | 623 | 235 | 17 | 508 | 210 | 23 | 0.89 |
| Jiang et al. (AA) | 2017 | 3,404 | 0 (100% African American) | 51.8 | 1543 | 121 | 1 | 1636 | 100 | 3 | 0.997 |

Supplementary Table S2: Detailed information of sensitivity analysis for the meta-analyses across all the combined studies.

| Removed Study Name | Odds Ratio (OR) | Lower Limit | Upper Limit | Z-Value | P-Value |
| --- | --- | --- | --- | --- | --- |
| Lang et al. (2007) | 1.248 | 1.046 | 1.490 | 2.936 | 0.003 |
| Wang et al. (2007) | 1.196 | 0.999 | 1.432 | 2.446 | 0.014 |
| Montag et al. (2008) | 1.255 | 1.050 | 1.499 | 2.979 | 0.003 |
| Landi et al. (2009) | 1.222 | 1.006 | 1.484 | 2.549 | 0.011 |
| Zhang et al. (2012) | 1.202 | 0.986 | 1.465 | 2.362 | 0.018 |
| Zhang et al. (2015) | 1.303 | 1.037 | 1.637 | 2.870 | 0.004 |
| Jiang et al (2017) EA | 1.206 | 1.005 | 1.448 | 2.513 | 0.012 |
| Jiang et al (2017) AA | 1.223 | 1.026 | 1.457 | 2.720 | 0.007 |

Supplementary Table S3: Detailed information of accumulative analysis across all the combined studies

| Published Year | Odds Ratio (OR) | Lower Limit | Upper Limit | Z-Value | P-Value |
| --- | --- | --- | --- | --- | --- |
| 2007 | 1.422 | 0.732 | 2.763 | 1.040 | 0.298 |
| 2008 | 1.113 | 0.638 | 1.939 | 0.377 | 0.706 |
| 2009 | 1.207 | 0.870 | 1.673 | 1.126 | 0.260 |
| 2012 | 1.259 | 0.983 | 1.612 | 1.827 | 0.068 |
| 2015 | 1.199 | 0.999 | 1.440 | 1.947 | 0.051 |
| 2017 | 1.229 | 1.031 | 1.464 | 2.306 | 0.021 |
