# Supplementary material for: Meta-analytic method reveal a significant association of theBDNF Val66Met variant with smoking persistence based on a large samples
Source: Pharmacogenomics J. 2019 Dec 2;20(3):398–407. doi: 10.1038/s41397-019-0124-y (PMC7253357; doi:10.1038/s41397-019-0124-y)
Supplement: Supplementary file 2 — Supplemental Figures [file 41397_2019_124_MOESM2_ESM.pptx]

## Slide 1
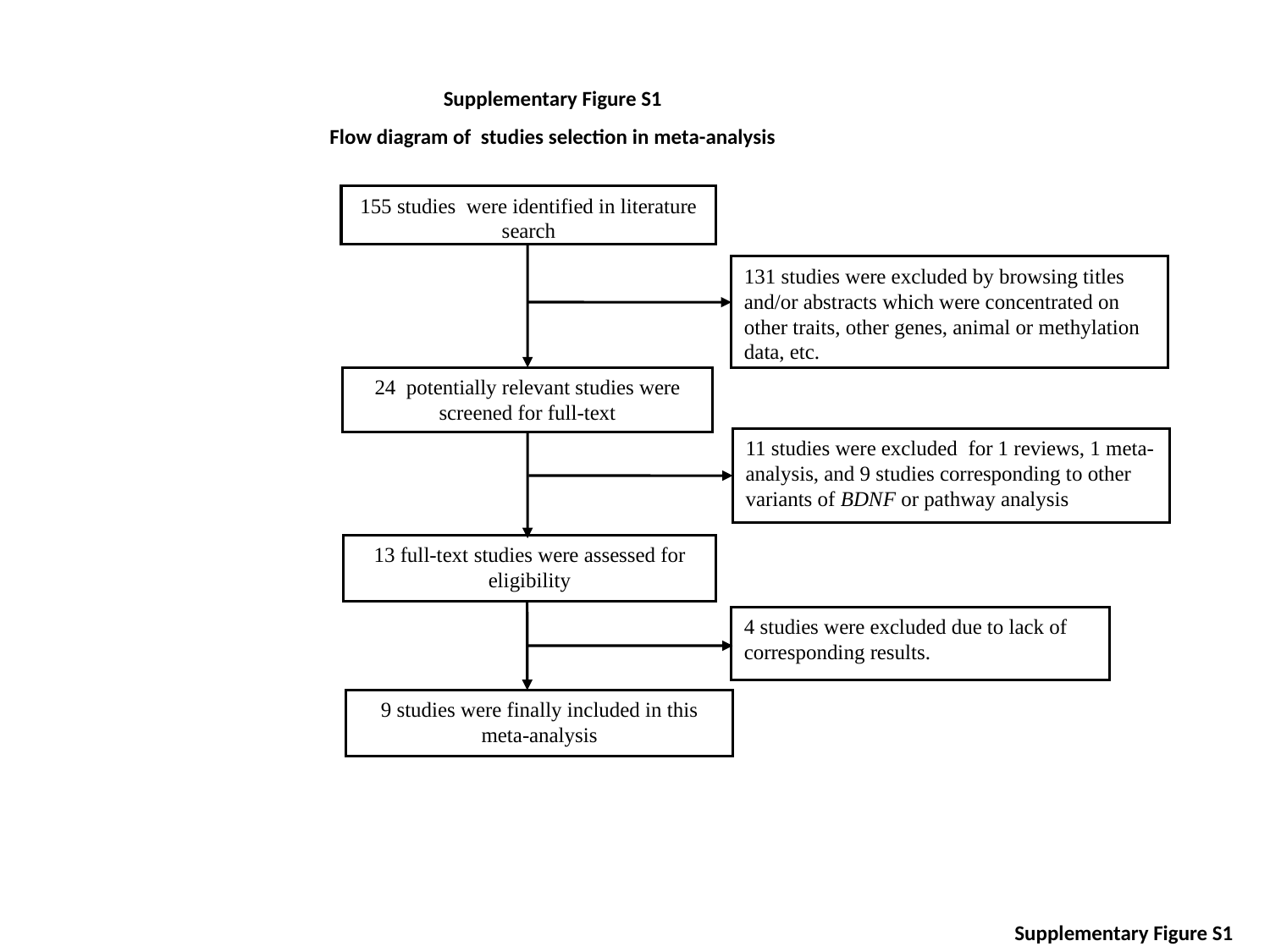

Supplementary Figure S1
Flow diagram of studies selection in meta-analysis
155 studies were identified in literature search
131 studies were excluded by browsing titles and/or abstracts which were concentrated on other traits, other genes, animal or methylation data, etc.
24 potentially relevant studies were screened for full-text
13 full-text studies were assessed for eligibility
4 studies were excluded due to lack of corresponding results.
9 studies were finally included in this meta-analysis
11 studies were excluded for 1 reviews, 1 meta-analysis, and 9 studies corresponding to other variants of BDNF or pathway analysis
Supplementary Figure S1

## Slide 2
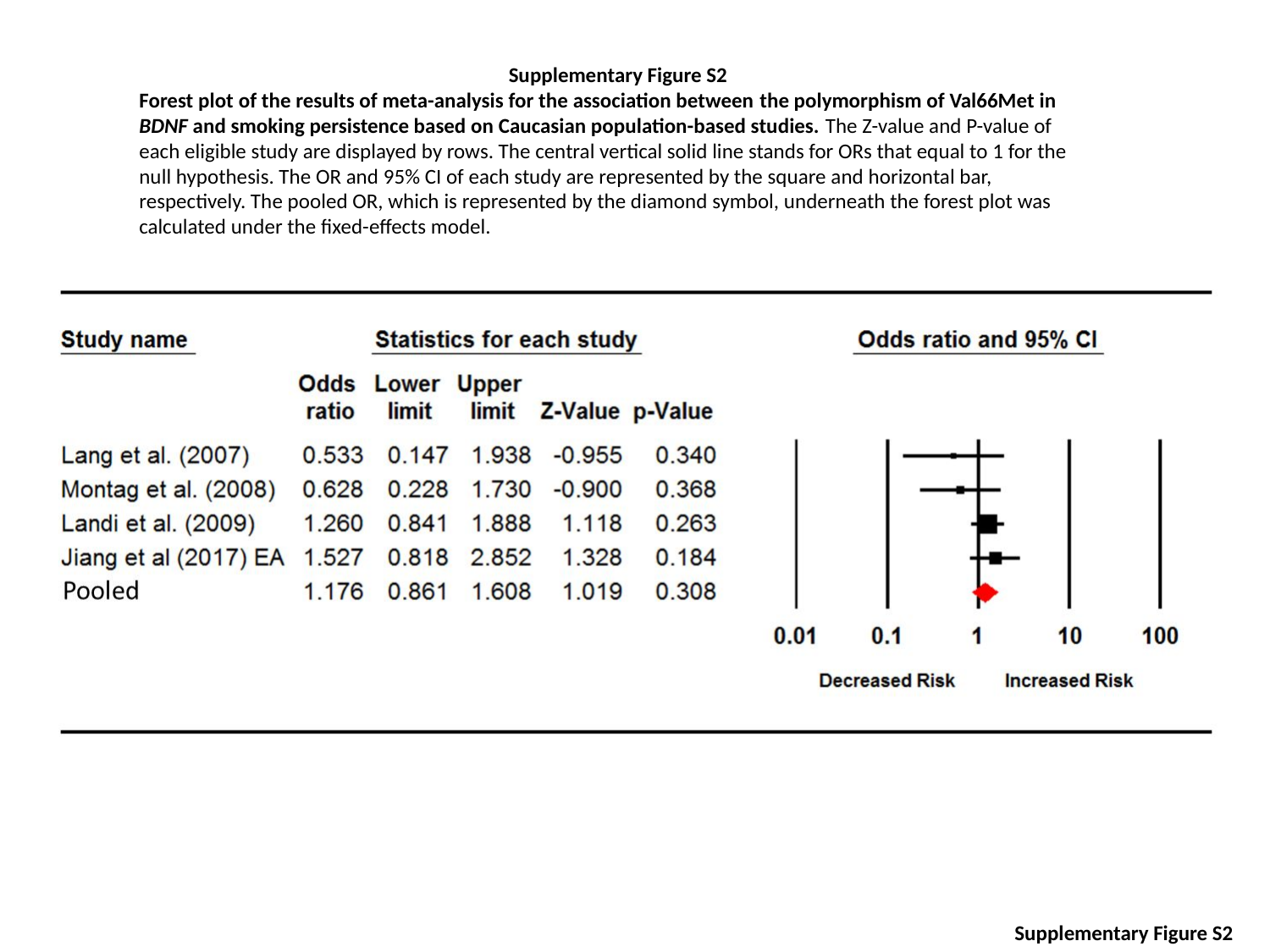

Supplementary Figure S2
Forest plot of the results of meta-analysis for the association between the polymorphism of Val66Met in BDNF and smoking persistence based on Caucasian population-based studies. The Z-value and P-value of each eligible study are displayed by rows. The central vertical solid line stands for ORs that equal to 1 for the null hypothesis. The OR and 95% CI of each study are represented by the square and horizontal bar, respectively. The pooled OR, which is represented by the diamond symbol, underneath the forest plot was calculated under the fixed-effects model.
Supplementary Figure S2

## Slide 3
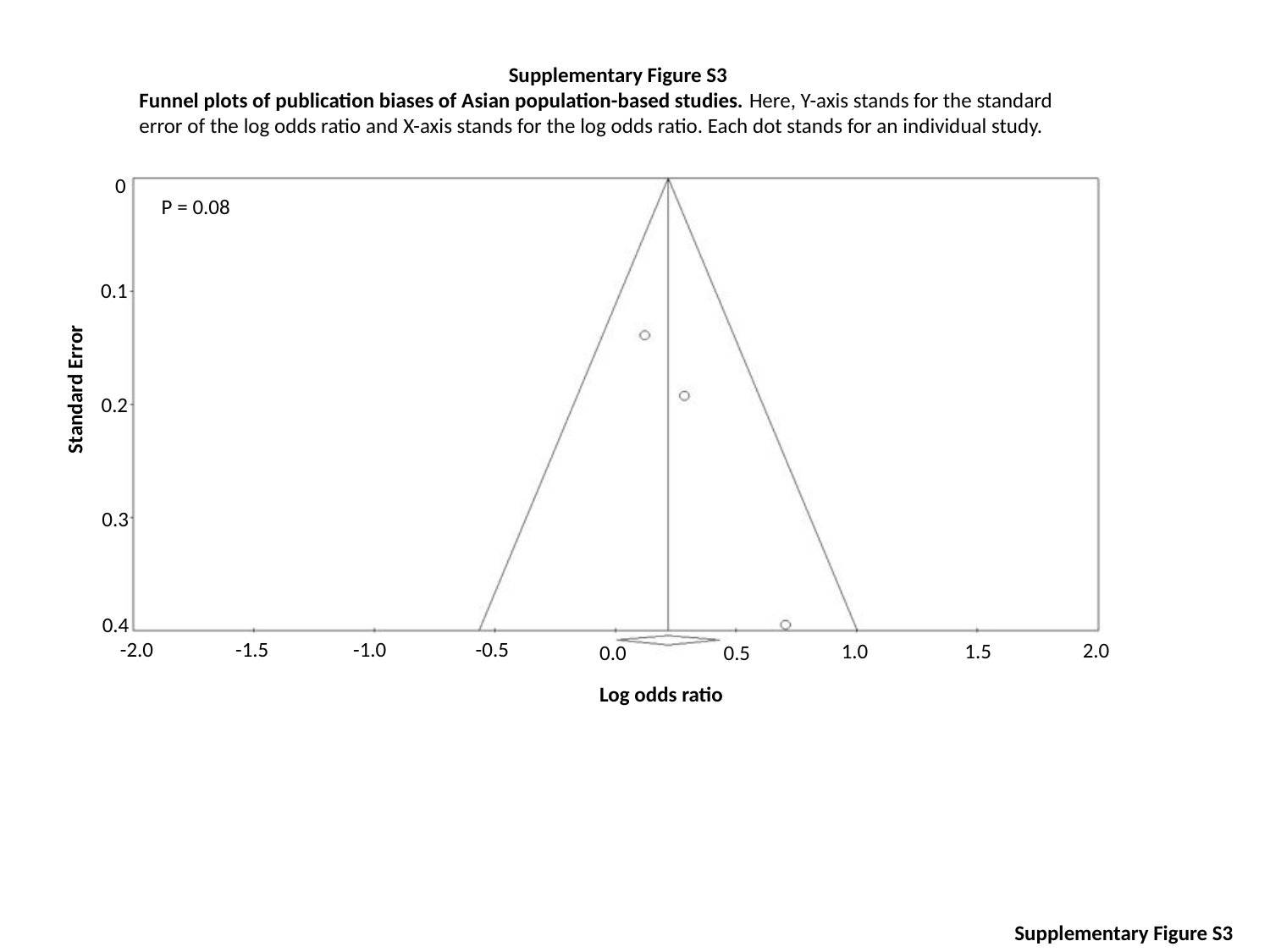

Supplementary Figure S3
Funnel plots of publication biases of Asian population-based studies. Here, Y-axis stands for the standard error of the log odds ratio and X-axis stands for the log odds ratio. Each dot stands for an individual study.
0
0.1
Standard Error
0.2
0.3
0.4
-0.5
-1.0
-2.0
-1.5
2.0
1.5
1.0
0.0
0.5
Log odds ratio
P = 0.08
Supplementary Figure S3

## Slide 4
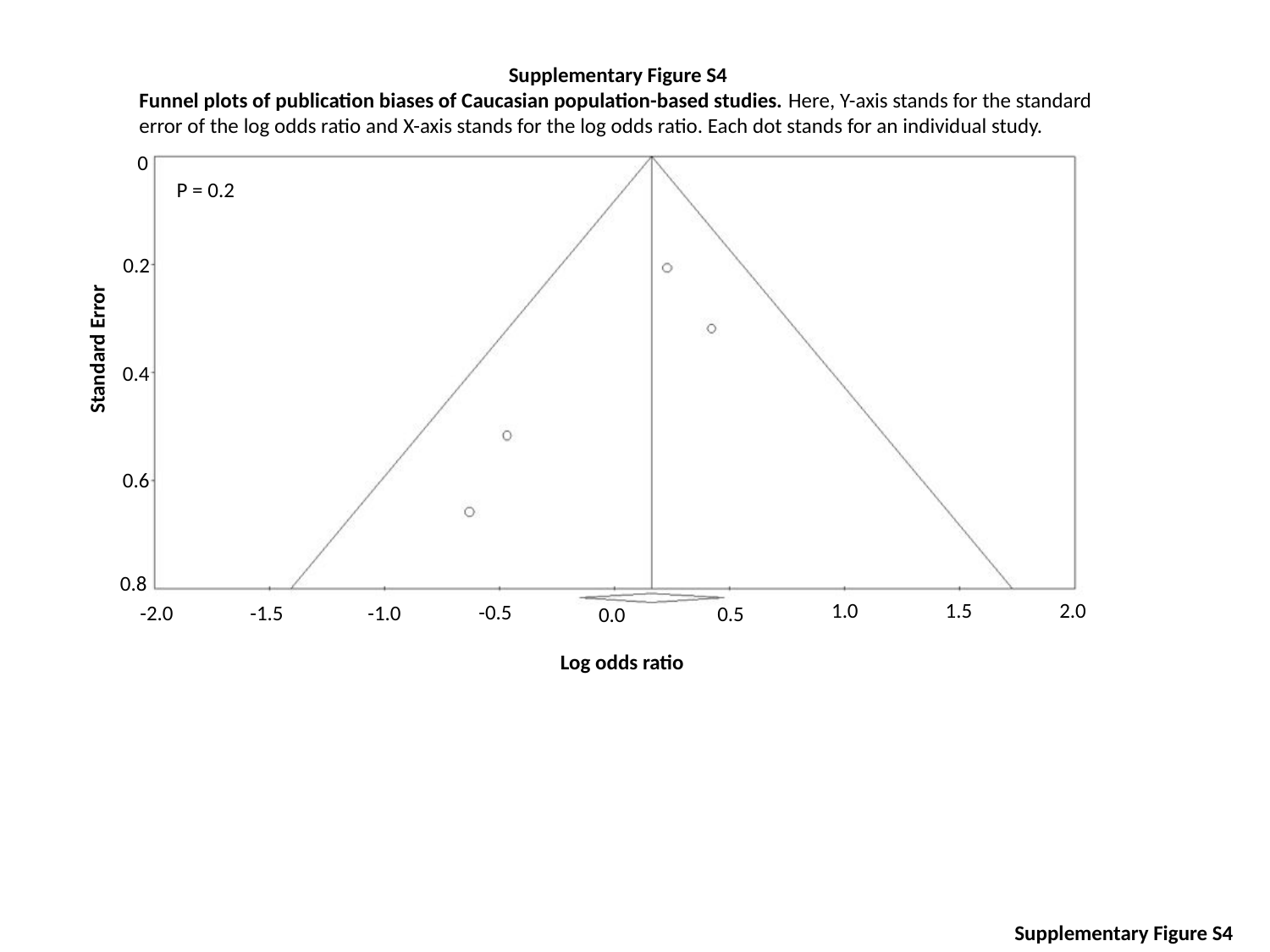

Supplementary Figure S4
Funnel plots of publication biases of Caucasian population-based studies. Here, Y-axis stands for the standard error of the log odds ratio and X-axis stands for the log odds ratio. Each dot stands for an individual study.
0
0.2
Standard Error
0.4
0.6
0.8
1.5
1.0
2.0
-0.5
-1.0
-2.0
-1.5
0.5
0.0
Log odds ratio
P = 0.2
Supplementary Figure S4
